# Surface Engineering in Materials (2nd Edition)

**DOI:** 10.3390/ma19102080

**Published:** 2026-05-15

**Authors:** Maria Richert

**Affiliations:** Faculty of Management, AGH University in Krakow, Krakow Gramatyka 10, 30-067 Krakow, Poland; mrichert@agh.edu.pl:

## 1. Introduction and Scope

Surface engineering is distinguished from the broader field of materials science by its specific research scope, focused on protecting, improving the quality, or enhancing the durability of surface and near-surface structures. Surface quality is usually the first element of finished products evaluated by consumers [1]. Therefore, great attention is paid to surface quality. Surfaces can be covered with various types of coatings, which, depending on the product’s intended use, serve a decorative purpose, provide protection against corrosion, or provide anti-wear protection. Surface modification can also be achieved through heat treatment [2], implantation [3], chemical interaction, and other technologies. Surfaces play a significant role in the functionality of products, due to characteristics such as hydrophobicity, hydrophilicity, extreme hardness, abrasion resistance, tightness, or, conversely, porosity, as well as smoothness or roughness, and a number of other features that determine their application [4].

The crucial role played by surfaces is reflected in the extensive literature on phenomena occurring on the surface and near-surface zones of materials [5]. Despite numerous studies, due to the importance of the issue and increasing material requirements, the search for increasingly better solutions and technologies for modifying surfaces and covering them with various types of coatings continues. An important element of this research is work on surface improvement that extends the range of applicability of materials by creating an external zone with properties other than those of the substrate [6]. There are numerous examples of practical applications of surface solutions, as well as in-depth studies of coatings, which fill the gaps in our understanding of the phenomena that generate surface modifications in materials. The research findings presented in the papers published in this Special Issue—“Surface Engineering in Materials” (2nd Edition)—address this topic.

## 2. Review of Published Articles

This Special Issue publishes five research reports from European countries concerning coating research and presents methods of modifying the surface of products manufactured using 3D additive technologies.

Investigating damage occurring in coatings faces several methodological challenges. One way to detect defects in coatings is through the use of acoustic emission. This topic is addressed in article [7]. Indentation tests on coatings were conducted using a Rockwell penetrator. The impact caused cracks to appear, most often observed around the indentation. As the cracks propagated, acoustic emission signals were generated. The energy released during this phenomenon is quantified as “impact energy” and is related to both the intensity and duration of the signal. Typically, these types of cracks are assessed through structural examination using optical or scanning electron microscopy. Acoustic emission signal measurements, on the other hand, can monitor crack propagation in real time. A correlation has been demonstrated between coating damage and acoustic emission (AE) signals in PVD coatings, which are difficult to analyze due to the very thin coating thicknesses of a few to several dozen micrometers. The presented research demonstrated a clear correlation between the cumulative acoustic emission energy and the area of coating delamination during Rockwell macro-indentation at high loads. The authors argue that this result demonstrates the possibility of finding an objective and effective alternative to existing methods for assessing adhesion, which is crucial for PVD coating applications.

Article [8] discusses the important role of coatings as lubrication zones. Friction is a factor leading to wear and damage. Therefore, there is a great need for solutions that reduce friction. The research presented in this article assessed the effect of modifying the chemical composition of an Al_2_O_3_ coating with tungsten disulfide IF-WS_2_ nanoparticles on abrasion. Inorganic Fullerene-like Tungsten Disulfide (IF-WS_2_) is a nanoparticle lubricant composed of nested, spherical layers that offer a superior friction reduction coefficient and high load-bearing capacity compared to conventional lubricants. The porosity of the anodic oxide layer is considered a functional feature, acting as a lubricant reservoir. The wear intensity of an Al_2_O_3_ coating with IF-WS_2_ nanoparticles in contact with a cast iron pin was investigated. The friction coefficient was analyzed in reciprocating motion without oil lubrication and under two load conditions. It was found that both the load on the lubrication node components and the coating modification had a significant effect on the friction coefficient. The use of IF-WS_2_-modified Al_2_O_3_ coatings reduced the coefficient of friction. It was also found that this coefficient decreased with increasing lubrication node load.

The creation of coatings through anodic oxidation is a technological process used, for example, in coating aluminum alloy products. This is an important stage of surface treatment, often determining the product’s quality. Understanding the mechanisms of this process is crucial for progress in aluminum surface modification. Oxidation conditions lead to a complex mechanism that includes not only electrochemical reactions but also physicochemical processes related to the presence of high temperature generated by micro-discharges of plasma in the oxide layer. One Special Issue paper deals with plasma electrolytic oxidation (PEO) as a method for producing oxide coatings on passivating metal substrates by electrochemical oxidation in aqueous electrolytes. During this process, a phenomenon called “soft sparking” can occur. This phenomenon has been reported primarily for aluminum and magnesium alloy substrates. Both the structure and properties of the coating on these metals can be partially altered by soft sparking. In this paper, the authors present a new perspective on soft sparking and propose a conceptual model for the formation of the oxide layer in PEO under AC conditions during the soft sparking state in particular.

The possibility of obtaining a protective ZrO_2_ coating on diamond particles was presented. The coating was intended to protect the diamond from oxidation and graphitization, enabling the diamond to be sintered at higher temperatures and lower pressures than its thermodynamic stability in atmospheric conditions. ZrO_2_ coatings were prepared by mechanically grinding diamond powders with zirconium powders, followed by heat treatment in an oxidizing atmosphere. Mixtures of diamond and 80 wt.% zirconium were sintered using the SPS method at temperatures of 1250 °C and 1450 °C. To stabilize the tetragonal structure of ZrO_2_, 3 mol.% Y_2_O_3_ was added to the zirconium prior to the grinding process. Coated diamond particles were found to be resistant to graphitization at high temperatures. However, the mechanical mixing of diamond powders with zirconium powders and the oxidation process do not guarantee uniform coating thickness. The composite ZrO_2_ material produced in this way, reinforced with diamond particles and then sintered using the SPS method at 1250 °C, may in the future be used as a tool material for stone cutting and drilling.

The latest advances in surface engineering are intelligent coatings. These are innovative coatings that spontaneously respond to external stimuli, thus providing added value beyond basic, passive functions such as decoration or surface protection. The triggers for this type of coating include heat, light radiation, mechanical induction, temperature, pressure, pH fluctuations, aggressive corrosive ions, and many other factors. Intelligent coatings include hydrophobic and superhydrophobic surfaces, which are inspired by nature. The design and production of superhydrophobic coatings inspired by nature involves creating materials with low surface energy and/or a rough surface texture. Innovative nanostructured technologies play a significant role in the production of this type of surface. Examples of intelligent coatings include antibacterial, antifouling, and conductive systems. Recent advances include superhydrophobic surfaces with self-cleaning and anti-icing properties. Nano-scale coatings produced by PVD are also noteworthy, as they have numerous applications in the metals industry thanks to well-developed coating deposition technology and the wide variety of materials that can be used to coat surfaces. Surface modification plays a significant role in 3D additive technologies. Products manufactured using this method are characterized by significant roughness and porosity. Various methods are currently being developed for modifying the surface of 3D products using methods such as etching, coating, micro- or nano-composition coating, and surface modification with a polymer brush. Metal parts also enable modification of surface wettability. Nanomaterial technologies combined with 3D technology often enable the achievement of unique properties unattainable using conventional technologies.

All articles presented in the Special Issue—“Surface Engineering in Materials” have citations that indicate interest in the topics discussed. This suggests that there is a significant need for information on phenomena, technologies, and applications related to surface and near-surface layers of materials, in part due to the significant demand and wide range of practical applications. Research in the field of surface engineering is becoming increasingly sophisticated. This is driven, on the one hand, by the pursuit of increasingly detailed and in-depth understanding of the phenomena occurring on surfaces and in subsurface layers during surface modification processes, and, on the other hand, by the increasing development of precise methods and tools enabling specialized research.

The surface engineering market is experiencing rapid growth, driven by demand for advanced coatings, smart surfaces, and functional processing methods in automotive, electronics, and aerospace. Key markets such as nanocoatings and smart surfaces are projected to experience significant growth, with smart surfaces expected to reach approximately $17.8 billion by 2030, driven by automotive and IoT applications. The global Smart Surfaces market is projected to grow from over $26 billion in 2024 to over $430 billion by 2030, reflecting rapid adoption, with a high ‘compound annual growth rate”, CAGR of 59.2%. CAGR is a financial metric used to measure the mean annual growth rate of an investment, revenue, or other business metric over a specified period longer than one year. Other estimates predict a rise to USD 17.82 billion by 2030 with a 15.1% CAGR. The Surface Treatment Chemical market is projected to see steady growth, with a CAGR of 5.9% from 2025 to 2032, expanding from USD 15.82 billion in 2025 [9,10].

## 3. Conclusions

The Special Issue “Surface Engineering in Materials” (2nd Edition) contains five publications addressing diverse issues related to coatings and layers deposited or produced on material surfaces, particularly metals. The overarching idea that unites the presented studies is a research area focused on the phenomena, technologies, and properties of the outer or subsurface layers of materials. Interest in this research area stems from its crucial role in the operational suitability of manufactured products. It is safe to say that virtually all products we encounter today have their surfaces modified or coated with special coatings. The pursuit of increasingly perfect surface coatings inspires the ever-increasing development of methods for manufacturing, controlling, and improving coatings, as well as research on the surface properties of materials. Recent advances in surface modification techniques offer a wide range of surface treatment applications. However, despite new advances aimed at increasingly precise operations, lower operating costs, and the availability of a wide range of processes, solutions require minimizing the environmentally harmful effects of process activities, in particular, developing principles for the widespread recycling and recovery of valuable raw materials contained in used coating materials.
